# Targeted Minimal Staff-to-Patient Ratios Are Unachievable – A Nationwide Survey in German ICUs During the COVID-19 Pandemic

**DOI:** 10.7759/cureus.15755

**Published:** 2021-06-19

**Authors:** Clemens Grimm, Steffen Dickel, Alexandra Sachkova, Maria Popp, Martin Golinksi, Falk Fichtner, Peter Kranke, Christian Seeber, Sven Laudi, Sebastian Voigt-Radloff, Onnen Moerer

**Affiliations:** 1 Department of Anesthesiology and Intensive Care Medicine, University Medical Center Goettingen, Goettingen, DEU; 2 Department of Anesthesiology, Intensive Care, Emergency and Pain Medicine, University Hospital Wuerzburg, Wuerzburg, DEU; 3 Department of Anesthesiology and Intensive Care Medicine, University Medical Center Leipzig, Leipzig, DEU; 4 Institute for Evidence in Medicine, Medical Center & Faculty of Medicine, University of Freiburg, Freiburg, DEU

**Keywords:** covid-19, icu, nurse-to-patient-ratio, physician-to-patient-ratio, nursing shortage, staffing

## Abstract

Introduction

Adequate staffing in the intensive care units (ICUs) is the most important factor to provide optimal care and ensure favorable outcomes in critically ill patients. Recently, the need for ICU beds has reached unprecedented levels and the management and treatment of critically ill patients has been in focus. The aim of the study was to assess the targeted and actual nurse-to-patient (NPR) and physician-to-patient ratios (PPR) regarding patients with and without COVID-19.

Methods

We conducted a nationwide online survey assessing the standard of care in German ICUs treating patients with COVID-19. We asked questions regarding targeted PPR and NPR and their implementation in daily clinical practice to heads of German ICU departments.

Results

We received 244 responses of which 171 were eligible for final analysis. Targeted median PPR ratio was 8 [interquartile range (IQR) = 4] and targeted NPR was 2 (IQR = 1). For COVID-19 patients, the median targeted PPR was 6 (IQR = 2) and the median targeted NPR was 2 (IQR = 0). Targeted PPRs were rarely met by 15.2% and never met by 3.5% of responding institutions. Targeted NPRs were rarely met in 32.2% and never in 5.3% of responding institutions.

Conclusion

In contrast to PPR, targeted NPRs were largely unattainable in German ICUs. Our results raise concern in view of studies linking worse outcomes in critically ill patients to suboptimal NPRs. This warrants further health policy efforts regarding optimal staffing in the ICU.

## Introduction

The intensive care treatment of critically ill patients places high demands on staffing. An adequate number of nurses and physicians per patient are substantial for individual outcome [1-4]. Major complications such as surgical bleeding, decubital ulcers, cardiac complications, occurrence of medication errors, unplanned extubations and prolonged ventilator weaning are associated with inadequate staffing in the ICU [1, 4-7].

The COVID-19 pandemic led to unprecedented adjustments in ICU organization and care processes. Bottlenecks in terms of availability of medical equipment were immanent and a serious problem in several countries, leading to severe strain. However, besides shortages in bed capacity or medical supplies, staff trained in the care of critically ill patients was the most limited resource.

The key figures that best determine an association between available professionals and patients are the physician-to-patient (PPR) and nurse-to-patient ratios (NPR) [8].

In the context of notorious staff shortage even before the SARS-CoV-2 pandemic, the problem was controversially discussed in daily politics. In Germany, this has been recently acknowledged by official authorities leading to a ministerial order ("Pflegepersonal Untergrenzenverordnung”, PpUGV) which has the intention to regulate a minimal standard of NPR on ICUs [9]. Accordingly, a minimum nurse-to-patient ratio of at least 1:2.5 at day and 1:3.5 at night is required [9]. However, the discrepancy between this political demand and the shortage of specialists in the ICU has remained unresolved and is a risk factor jeopardizing a high standard of care.

The current COVID-19 crisis highlights this familiar policy problem in a new, pandemic situation. Patients infected with SARS-CoV-2, like all infectious and isolated patients per se, require a much higher level of support than non-contagious patients, resulting in an unprecedented workload for medical personnel [10].

Our aim was to answer two questions: first, the targeted PPR and NPR were queried by interviewing heads of ICU departments. The second question focused on the actual implementation of these self-proclaimed standards in daily practice during the COVID-19 pandemic.

## Materials and methods

The survey was generated within a nationwide network established for clinical research in this on-going pandemic (German COVID-19-evidence ecosystem, CEOsys), funded by national authorities (Federal Ministry of Education and Research, BMBF) [11]. The interdisciplinary study team consisted of 11 experts as part of the CEOsys network. Our research strategy is described as follows: We identified ICUs under the above-mentioned criteria (COVID-19 and non-COVID-19) via the German Interdisciplinary Association for Intensive Care and Emergency Medicine (DIVI) and used their email register to contact ICUs in Germany. Together with the email we invited the responsible head of ICU departments or responsible physician to answer the following question by an online survey: (Q1) “Please indicate the acceptable number of non-COVID-19 patients treated by one physician in your institution (Self-assessed target for physician-to-patient ratio). (Q2) “Please indicate the acceptable number of COVID-19-patients treated by one physician in your institution”. (Q3) “Please indicate the acceptable number of non-COVID-19 patients treated by one nurse in your institution (Self-assessed target for nurse-to-patient ratio)”. (Q4) “Please indicate the acceptable number of COVID-19-patients treated by one nurse in your institution”. A quality assessment regarding the fulfillment of self-proclaimed targets was addressed by the following request: “Please estimate the fulfillment of your self-proclaimed target ratios (Q1-Q4) using the following four options. Our institution is i) always, ii) often, iii) rarely or iv) never able to fulfill the previously mentioned targets.” There was no possibility for the study team to track individual responses (anonymized survey). To avoid multiple responses, such as double input by head of departments or their leading physicians, we identified each institution by a code automatically generated by zip code, telephone number, floor of the ICU and hospital location. Participation was voluntary. Randomization or alteration of the items’ order of appearance was not carried out. Potential participants were informed about the time duration, data privacy, the investigators, and the purpose of the questionnaire according to the CHERRIES criteria [12]. IP-checks, log files or cookies were not used. Ethical approval was waived after a formal request to the University of Würzburg’s IRB [219/20-am], since no personalized patient data was obtained during our study.

The survey was created by using the SoSci survey online tool. Statistical analysis was performed using Microsoft Excel (Microsoft® Corp., Redmond, WA) and IBM SPSS version 27.0.0.0 (IBM Corp., Armonk, NY). Python version 3.8.2 (Python Software Foundation, Wilmington, DE) in a Jupyter notebook including the seaborn sublibrary was used for data visualization. We used the chi-square test to show differences between professional groups and calculated median and interquartile range (IQR) values. P-values less than .05 were considered significant.

## Results

**Figure 1:**
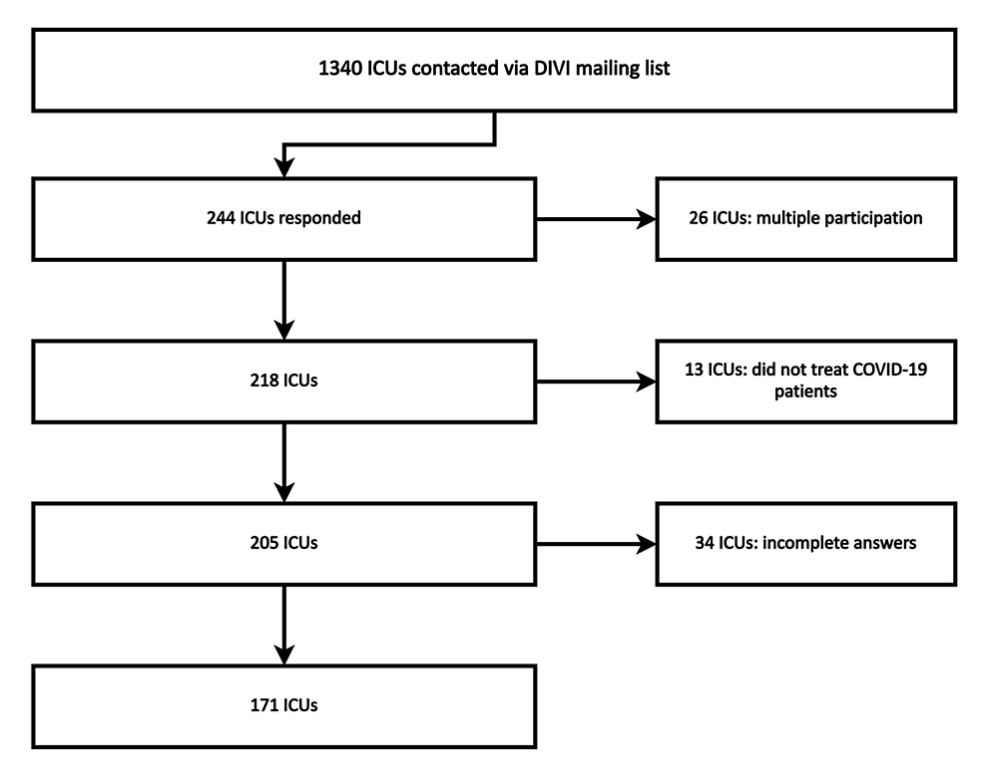
Flow-chart of the study procedure, drop-out rates and reasons. ICU: Intensive care unit; COVID-19: Coronavirus disease 2019, DIVI: Deutsche Interdisziplinäre Vereinigung für Intensiv- und Notfallmedizin (German Interdisciplinary Association for Intensive Care and Emergency Medicine).

During the three-week processing period, we intended to contact 1340 ICUs from a register of the German Society of Intensive Care and Emergency Medicine (DIVI). Figure 1 represents this process: Two-hundred forty-four ICUs responded, 16.6% of the respondents did not answer all questions and 6.0% of all answering ICUs did not treat COVID-19 patients and were excluded. A total of 171 ICUs qualified for full validation (Figure 1). The majority of evaluated ICUs were based within medium-sized hospitals with 200-1000 beds (53%), 32% were hospitals with more than 1000 beds and 13% with less than 200 beds.

Four questions were asked to the heads of ICU departments or the physicians responsible for allocation of staff. The answers regarding staff-to-patient ratio in each institution are presented in Table 1. The indicated and targeted “ideal” PPR for non-COVID-19 is eight patients and six patients for the treatment of COVID-19 patients. The variation between ICUs, represented in the IQR was higher for non-COVID-19 patients (Table 1). In contrast to PPR the ratio was significantly lower regarding NPR: One nurse is planned to treat two ICU patients. The commitment to this “ideal” and self-pronounced ratio is even clearer in the COVID-19 pandemic with no variation (IQR = 0, Table 1).

**Table 1:** Targeted physician-to-patient and nurse-to-patient ratios for ideal patient care as stated by heads of departments (n = 171). Q: Question; COVID-19: Coronavirus-disease 2019; IQR: Interquartile range.

Question	Target: Physician-to-patient ratio	Median (IQR)
Q1	Non-COVID-19	8 (4)
Q2	COVID-19	6 (2)
Question	Target: Nurse-to-patient-ratio	Median (IQR)
Q3	Non-COVID-19	2 (1)
Q4	COVID-19	2 (0)

**Figure 2:**
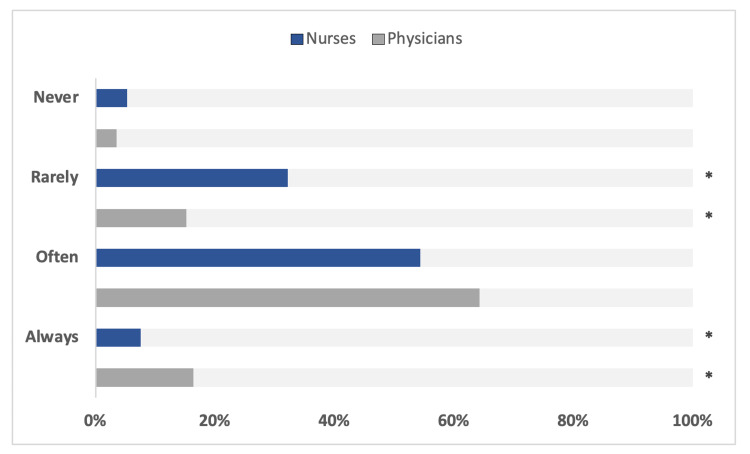
Results from the survey regarding self-assessment of ICU head of departments to fulfill self-pronounced personnel target ratios. A total of 171 ICUs are presented. Significant differences for p < 0.05 are marked with *.

The survey focused on target achievement by self-assessment of heads of ICUs. Table 2 and Figure 1 represent the results regarding the fulfillment of self-stated targets. Above 80% of the consulted ICUs are “always” or at least “often” able to fulfill their own requirements regarding PPR, which is significantly higher compared to the fulfillment of NPR targets (Figure 2). Conversely, 37.5% of ICUs are unable to allocate enough nursing staff to the most critical patients (Table 1 and Figure 2). This points to a staffing shortage in nurses but to a lower extent in physicians.

**Table 2:** Self-assessment of heads of ICUs in Germany (n = 171) regarding the fulfillment of adequate, self-pronounced physician-to-patient (PPR) and nurse-to-patient (NPR) ratios.

Fulfillment of targets	PPR	NPR	p-Value
Always, n (%)	28 (16.4%)	13 (7.6%)	<0,05
Often, n (%)	110 (64.3%)	93 (54.4%)	0,08
Rarely, n (%)	26 (15.2%)	55 (32.2%)	<0,01
Never, n (%)	6 (3.5%)	9 (5.3%)	0,59

## Discussion

The purpose of this study was to identify self-pronounced staff-to-patient ratios by a structured, online-based approach followed by self-assessment of whether these ratios can be achieved.

A targeted PPR of 1:8 for non-COVID-19 patients and 1:6 for COVID-19 patients and a minimum NPR of 1:2 regardless of COVID-19 was reported. While the majority of institutions are able to recruit physicians to achieve their targets, the bottleneck is the nursing staff. Our results underline an important issue in the German health care system. The expectation to the standard-of-care in terms of available ICU personnel is high. However, the reality shows that self-pronounced targets of the responsible ICU management are unachievable.

Beyond all doubt an adequate staff-to-patient ratio is crucial in intensive care medicine [1, 7, 13]. This evidence has been acknowledged by the ministry of health in Germany (BMG) and by guideline recommendations of the German Interdisciplinary Association for Intensive Care and Emergency Medicine (DIVI) [9, 14]. It is thus unsurprising that the heads of ICU departments in our survey committed themselves to a minimal quality standard of patient care, not only in terms of NPR but also PPR (Table 1).

During the COVID-19 pandemic, with a significant percentage of critically ill patients requiring intensive care, a substantial increase in available ICU capacity without additional staffing can only be achieved by reducing the influx of non-COVID patients. However, because a large proportion of necessary ICU admission cannot be avoided without putting patients at risk, for example by reducing deferrable major surgical procedures [15], additional bed capacity has been created in many places. In Germany, these additional capacities could only be partially covered by the primary available ICU staff. Tiered staffing models, just-in-time training for non-ICU clinicians, designated treatment teams, and deployment of trainees [15] were employed in this regard. However, as revealed by our survey, these measures were only partly successful in maintaining the increased needs.

While most studies focused on NPR, data regarding ideal PPR is limited. For example, one study addressed the question of an ideal ratio of physician-to-ICU beds. The authors found that ICU length of stay is significantly prolonged if the ratio reaches 1:15 [3]. Another study showed that an unfavorable PPR greater than 1:7.5 increases ICU mortality [16]. How can we interpret the reported target PPRs of 1:8 and 1:6, respectively? Those ratios can be considered as an appropriate approach for the management of critically ill patients and reflect an adjusted ratio in the management of isolated and complex COVID-19 patients. They are in line with current recommendations and guidelines [3, 14, 16]. However, the high PPR variance in the above-mentioned studies from 1:7.5 to 1:15 suggests that additional factors are even more important than statistical heads per patient. From our survey we cannot answer this question to what degree skills such as clinical experience or the level of training influence the outcome. However, one may assume that soft skills are extremely relevant. Nevertheless, the majority of ICUs are at least able to provide enough physicians to fulfill their self-pronounced targets (Table 2).

An important finding of our study is the identification of a discrepancy when comparing physicians and nurses. In summary, 37.5% of ICUs in our survey are "never" or "rarely" able to achieve the targeted NPR (Table 2 and Figure 1). This can have dramatic consequences. Intensive care medicine is highly dependent on therapy by nurses, who are largely responsible for therapeutic measures such as weaning, patient mobilization, or hygiene measures. From this point of view, it is reasonable to assume that many patients may not receive optimal treatment. One may critically ask whether a self-reported PPR ratio of 1:6 or even less is irrelevant when the most important clinical team member, the nurse, is absent. A new metric that might better reflect the division of labor in ICU teams could be a physician-to-nurse-to-patient ratio in the ICU.

Although our survey is not representative, which is clearly a limitation of this study, the response of 171 ICUs highlights a widely discussed problem in German public health. Furthermore, since 49% were larger hospitals with more than 600 beds, we cannot generalize our results but we are worried that the situation in smaller or non-responding hospitals might be even worse.

## Conclusions

Our survey suggests that the discrepancy between desired and actual staffing levels is significant, particularly among nurses. Because quality of care and complication rates are significantly correlated with NPR, it is reasonable to suspect that this shortage is leading to a reduction in quality. With more than one-third of reporting ICUs failing to achieve adequate nurse staffing during the COVID-19 pandemic, there is a risk that significant reductions in quality might have occurred. Our results warrant further health policy efforts regarding optimal staffing in the ICU.
